# Identification of phenomic data in the pathogenesis of cancers of the gastrointestinal (GI) tract in the UK biobank

**DOI:** 10.1038/s41598-024-52421-9

**Published:** 2024-01-23

**Authors:** Shirin Hui Tan, Catherina Anak Guan, Mohamad Adam Bujang, Wei Hong Lai, Pei Jye Voon, Edmund Ui Hang Sim

**Affiliations:** 1 Clinical Research Centre, Sarawak General Hospital, Ministry of Health Malaysia, Jalan Hospital, 93586 Kuching, Sarawak Malaysia; 2 Faculty of Resource Science and Technology, Universiti Malaysia Sarawak, 94300 Kota Samarahan, Malaysia; 3 Department of Radiotherapy, Oncology and Palliative Care, Sarawak General Hospital, Ministry of Health Malaysia, Jalan Hospital, 93586 Kuching, Sarawak Malaysia

**Keywords:** Cancer, Molecular biology, Biomarkers, Molecular medicine, Oncology

## Abstract

Gastrointestinal (GI) cancers account for a significant incidence and mortality rates of cancers globally. Utilization of a phenomic data approach allows researchers to reveal the mechanisms and molecular pathogenesis of these conditions. We aimed to investigate the association between the phenomic features and GI cancers in a large cohort study. We included 502,369 subjects aged 37–73 years in the UK Biobank recruited since 2006, followed until the date of the first cancer diagnosis, date of death, or the end of follow-up on December 31st, 2016, whichever occurred first. Socio-demographic factors, blood chemistry, anthropometric measurements and lifestyle factors of participants collected at baseline assessment were analysed. Unvariable and multivariable logistic regression were conducted to determine the significant risk factors for the outcomes of interest, based on the odds ratio (OR) and 95% confidence intervals (CI). The analysis included a total of 441,141 participants, of which 7952 (1.8%) were incident GI cancer cases and 433,189 were healthy controls. A marker, cystatin C was associated with total and each gastrointestinal cancer (adjusted OR 2.43; 95% CI 2.23–2.64). In this cohort, compared to Asians, the Whites appeared to have a higher risk of developing gastrointestinal cancers. Several other factors were associated with distinct GI cancers. Cystatin C and race appear to be important features in GI cancers, suggesting some overlap in the molecular pathogenesis of GI cancers. Given the small proportion of Asians within the UK Biobank, the association between race and GI cancers requires further confirmation.

## Introduction

The prevalence of gastrointestinal (GI) tract cancers are a significant public health issue worldwide, given their substantial contribution to the overall incidence and mortality rates of cancer on a global scale. GI tract cancers accounted for 26.3% of the total 4.8 million cancer cases and 35.3% of the 3.4 million cancer-related deaths in the year 2018^1^; encompassing the oesophagus, stomach, liver, pancreas, small intestine, colon, and rectum, are commonly referred to as GI tract cancers^2^. In addition, Arnold et al.^3^ reported that the predominant GI malignancies include colorectal cancer (10.2%), stomach cancer (5.7%), liver cancer (4.7%), oesophageal cancer (3.2%), and pancreatic cancer (2.5%). GI tract cancers present with varying clinical characteristics and risk factors, intimately linked with lifestyle decisions and pre-cancerous ailments^3^; inclusive phenotypic information across multiple levels, encompassing clinical, molecular, and cellular aspects.

Phenotypic data, an integral component of phenomics, provides a comprehensive understanding of observable traits and characteristics, contributing to a holistic analysis of biological systems; whilst phenomics involves quantifying the phenome, a set of observable characteristics encompassing physical, chemical, and biological traits in individuals and populations^4,5^. These traits arise from intricate interplays among genetic factors, environmental conditions, dietary influences, and symbiotic microorganisms^6^. Phenomic studies, in contrast to traditional biomedical research, exhibit unique features such as meticulous standardization in measurements, data management and analysis; relying on extensive big data, encompassing multi-dimensional and well-organized datasets^7^. In the domain of cancer, comprehending the entire spectrum of phenotypic irregularities associated with malignancies, including the intricate details revealed by biomarkers, is imperative for advancing our knowledge of carcinogenesis, especially the mechanisms underlying the relationships among phenome, genome and environmental impact.

Following the completion of the Human Genome Project, comprehensive explorations into the human phenome have become crucial, forming a foundational framework for deciphering the intricacies of human health codes, especially the complex relationships among the phenome, genome and environmental influences^7^. Previous research has documented the use of phenomic data to investigate the correlation between environmental factors, such as diet and lifestyle, and the development of various cancers^8–12^. Furthermore, different toolkits associated with cancer phenomics were examined^13–16^. In the realm of cancer research, there is currently a limited body of knowledge regarding the exploration of diverse cancer types using phenomics data sourced from extensive datasets that encompass comprehensive clinical information. This scarcity is particularly evident when considering GI cancer, where the understanding of the complex interplay between phenotypic characteristics and the underlying molecular mechanisms remains relatively under-explored. Given this research gap, the UK Biobank emerges as an unparalleled resource, presenting an extraordinary opportunity to address the gap in our understanding of the involvement of phenomic data in GI cancer research.

The UK Biobank is a prospective cohort study of considerable magnitude that has enlisted more than 500,000 individuals between the age of 40 and 69 years from various regions of the UK during the period of 2006 to 2010. The extensive sample size and comprehensive data collection of phenotypic and genotypic data enable the examination of intricate associations between socio-demographic factors, blood chemistry, anthropometric measurements, and lifestyle of participants, thereby facilitating the development of more efficacious prevention and treatment approaches^17,18^. Within the national cancer registry, UK Biobank participants have contributed to a large accumulation of data comprising over 43,000 newly reported cancer cases up to the present. The UK Biobank is uniquely equipped to facilitate research into the factors that contribute to the onset of disease. It facilitates the identification of risk factors that increase or decrease the likelihood of developing specific diseases, as well as the precise quantification of these associations' magnitude. In addition, the substantial diversity observed in the intensity of these associations across various demographic, socioeconomic, and lifestyle characteristics provides an opportunity to assess the applicability of these associations to substantial subgroups of the population^19,20^.

Growing evidence highlights the importance and pressing need for utilizing phenomics in the examination of diseases^21–24^. Given the limited research on GI phenomics, this study was undertaken to explore the correlation between GI cancers and phenomic characteristics within the UK Biobank cohort. The UK Biobank offers a comprehensive range of socio-demographic, anthropometric, and biological markers, including blood and urine biomarkers, making it a valuable resource for this investigation. It is anticipated that the outcomes of this study will contribute to a more profound understanding of the multi-omics composition of patients, complemented by clinical data. This understanding, in turn, is expected to facilitate the identification of diagnostic, prognostic, and predictive biomarkers. Furthermore, the insights gained from this research endeavor hold the potential to unveil effective pathways for the personalized treatment of a diverse range of targeted diseases.

## Methods

### Study design and participants

The UK Biobank is a prospective cohort study with the aim of investigating how various diseases are caused by genetic, environmental, and lifestyle factors. Every participant in the UK Biobank provided informed consent upon enrolment, granting permission for the sharing of anonymized data with authorized researchers. Participants retained the right to withdraw their consent for data sharing at any point during their participation. All participants were registered with the National Health Service (NHS) in the United Kingdom. Participants completed a self-administered touchscreen questionnaire regarding their sociodemographics, lifestyle behaviours, medical history, and medication use during the initial recruitment session. They also underwent physical measurements such as weight, height, waist circumference, and hip circumference. Detailed information of the UK Biobank has been reported previously^25^. Since its establishment in 2006, a total of 502,369 subjects aged 37–73 years were recruited between 2006 and 2010 and followed up since then^19^ until the date of the first cancer diagnosis, date of death, or the end of follow-up on December 31st, 2016, whichever occurred first. Access to the UK Biobank data was applied and approved (Application number 96759). This study was also approved by the Malaysia Medical Research and Ethics Committee (NMRR ID-23-00931-SPO).

**Figure 1:**
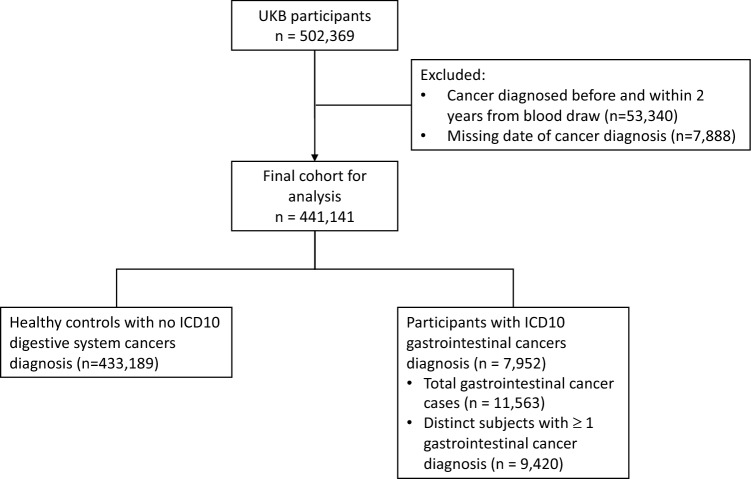
Flow diagram for exclusion and inclusion.

Figure 1 illustrated the flow diagram for exclusion and inclusion in this study. After taking into consideration the exclusion criteria, we included 433,189 controls for our analyses. Controls were participants who did not have a record of ever being diagnosed with cancer according to the 10th Revision of the International Classification of Diseases (ICD-10). As for the cases, we included incident cancer cases who had GI cancers as coded using the ICD-10. GI cancers referred in this study included C15 oesophageal cancer, C16 gastric cancer, C17 small intestine cancer, C18 colon cancer, C19 rectosigmoid junction cancer, C20 cancer of rectum, C21 cancer of anus and anal canal, C22 liver cancer, C23 gallbladder cancer, C24 cancer of other and unspecified parts of biliary tract, C25 pancreatic cancer and C26 cancer of other digestive organs. We excluded participants with any GI cancers diagnosed within two years from recruitment (n = 55,340) to account for reverse causation, and those with missing date of cancer diagnosis (n = 7888). Finally, we included 7952 participants who had GI tract cancers as coded using the ICD-10.

### Phenomic analysis

Sociodemographic characteristics (gender, age, and race) and lifestyle factors (smoking, alcohol drinking and physical activity) were collected during baseline assessment. Smoking status and alcohol drinking status were categorized as Never, Previous or Current smoker or alcohol drinker respectively, as recorded in the UK Biobank and reported previously^26,27^.Townsend deprivation index score which reflected the socioeconomic status were calculated for each participant based on the postcodes of residence. Age was calculated based on age from date of birth and baseline assessment visit. Physical activity was collected based on number of days that the participants had moderate or vigorous activity for at least 10 min. As part of the research interest is to investigate the differences between race in cancer occurrence, we also explored race as categorized by White (British, Irish, White and any other White background) versus Asians (Chinese, Indian, Pakistani, Bangladeshi and any other Asian background).

Anthropometric measurements including height, body weight, waist circumference and hip circumference were taken by trained nurses during the baseline assessment visit^28^. Body mass index (BMI) was calculated as weight/height^2^. We then categorized the BMI based on the WHO BMI classification^29^.

Biological markers were obtained from serum and urine samples. Efforts were put in by the UK Biobank to minimise systematic and random errors in the biomarker assays, including blood and urine samples analyses^30,31^. We included biomarkers related to glucose control (glycated hemoglobin (HbA1c)), cardiovascular health (Apolipoprotein A, Apolipoprotein B, C-reactive protein (CRP), lipoprotein(a), high density lipoprotein (HDL) cholesterol, low density lipoprotein (LDL) cholesterol, triglyceride, total cholesterol), renal profile (creatinine, cystatin C, total protein, urate and urea), liver profile (alanine transferase (ALT), alkaline phosphatase (ALP), aspartate transferase (AST), gamma-glutamyl transferase (GGT), albumin, total bilirubin, direct bilirubin), hematological parameters (basophil count, eosinophil count, erythrocyte count, hemoglobin concentration, leukocyte count, lymphocyte count, monocyte count, neutrophil count, platelet count), hormones (insulin like growth factor 1 (IGF-1), oestradiol, testosterone, sex hormone binding globulin (SHBG)), and bone-related markers (ionized calcium, phosphate, 25-hydroxyvitamin D (25(OH)D) and rheumatoid factor).

Blood and/or urine samples were available for all the participants. Participants without any of the blood and urine biomarkers available were not included in this study. However, there were missing data for the variables included in this study. No imputation was performed for the missing data. For the analysis of each individual parameter, participants with missing data were excluded. Due to the large number of variables involved, information on the missing data is available upon request.

### Statistical analyses

This study aimed to determine the risk factors for GI cancers. The risk factors explored consisted of 59 parameters as mentioned previously. The outcome was defined as the diagnosis of incident total GI cancers (based on ICD-10 C15-C26) and each individual diagnosis of GI cancers. Descriptive statistics were used to describe the characteristics of these variables based on each individual GI cancers, total GI cancers and healthy controls (Table 1).

**Table 1:** Baseline characteristics of the study population in the UK Biobank.

Characteristics^a^	Colorectal cancer (N = 5436)	Pancreatic cancer (N = 1286)	Esophageal cancer (N = 1146)	Stomach cancer (N = 878)	Liver cancer (N = 755)	Other GI cancers^b^ (N = 1027)	Total GI cancers (N = 7952)	Participants without GI cancers (N = 433,189)
Sociodemographic								
Age at recruitment (years)	60.36 (6.72)	61.06 (6.32)	61.09 (5.98)	60.839 (6.51)	61.10 (6.10)	59.95 (6.80)	60.37 (6.67)	56.00 (8.12)
Age at cancer diagnosis	67.47 (7.10)	68.54(6.76)	68.516 (6.52)	68.069 (6.85)	68.62 (6.55)	67.16 (7.19)	67.86 (7.10)	–
Male sex (%)	3082 (56.70)	705 (54.82)	853 (74.43)	630 (71.75)	483 (63.97)	488 (47.52)	4615 (58.04)	200,674 (46.30)
Townsend deprivation index	− 1.42 (3.08)	− 1.19 (3.20)	− 0.95 (3.28)	− 0.904 (3.24)	− 0.80 (3.41)	− 1.02 (3.32)	− 1.24 (3.17)	− 1.28 (3.10)
Race (%)								
White	5245 (97.11)	1228 (95.86)	1120 (98.42)	830 (95.07)	716 (95.47)	978 (95.60)	7645 (96.67)	405,842 (94.21)
Asians	57 (1.06)	13 (1.01)	8 (0.70)	13 (1.49)	12 (1.60)	14 (1.37)	93 (1.18)	10,869 (2.50)
Black or Black British	43 (0.80)	20 (1.56)	3 (0.26)	15 (1.72)	8 (1.07)	5 (0.49)	74 (0.94)	7277 (1.69)
Mixed	32 (0.59)	9 (0.70)	3 (0.26)	6 (0.69)	3 (0.40)	16 (1.56)	44 (0.56)	2669 (0.62)
Other racial groups	27 (0.50)	11 (0.86)	4 (0.35)	9 (1.03)	11 (1.47)	10 (0.98)	53 (0.67)	4118 (0.96)
Anthropometric measurements								
Height, cm	169.74 (9.16)	169.21 (9.03)	171.23 (8.53)	171.08 (8.75)	170.19 (9.06)	168.60 (9.16)	169.74 (9.15)	169.00 (9.31)
Weight, kg	80.83 (16.17)	81.43 (16.49)	84.61 (17.86)	84.41 (16.76)	84.58 (18.27)	80.39 (17.4)	81.62 (16.89)	78.10 (16.0)
Body mass index, kg/m^2^	27.98 (4.75)	28.29 (4.97)	28.76 (5.34)	28.77 (5.05)	29.12 (5.62)	28.18 (5.15)	28.24 (5.01)	27.40 (4.79)
Waist circumference, cm	93.65 (13.69)	94.07 (13.71)	97.69 (14.39)	97.01 (13.89)	97. 45 (15.05)	92.80 (14.50)	94.35 (14.21)	90.30 (13.50)
Hip circumference, cm	104.11 (9.01)	104.77 (9.92)	104.57 (9.97)	104.44 (9.57)	105.97 (10.67)	104..40 (20.28)	104.45 (9.60)	103.00 (9.22)
Lifestyle factors								
Smoking status (%)								
Never	2527 (46.69)	584 (15.64)	380 (33.39)	341 (39.15)	297 (39.49)	483 (47.12)	3580 (45.19)	238,595 (55.40)
Previous	2316 (42.79)	498 (13.34)	522 (45.87)	400 (45.92)	337 (44.81)	422 (41.18)	3335 (42.10)	146,206 (33.90)
Current	569 (10.51)	199 (5.33)	236 (20.74)	130 (14.93)	118 (15.69)	120 (11.71)	1007 (12.71)	45,966 (10.70)
Alcohol drinking status (%)								
Never	197 (3.63)	61 (4.75)	43 (3.77)	38 (4.34)	43 (5.72)	57 (5.56)	336 (4.23)	19,185 (4.44)
Previous	206 (3.80)	55 (4.29)	77 (6.76)	52 (5.94)	40 (5.32)	36 (3.51)	358 (4.50)	14,911 (3.45)
Current	5017 (92.56)	1167 (90.96)	1020 (89.47)	786 (89.73)	669 (88.96)	932 (90.93)	7237 (91.02)	397,648 (92.10)
Physical activity								
Vigorous physical activity, days/week	1.72 (1.93)	1.80 (1.97)	1.76 (2.06)	1.74 (2.05)	1.58 (1.96)	1.68 (1.94)	1.73 (1.96)	1.87 (1.92)
Moderate physical activity, days/week	3.65 (2.32)	3.70 (2.30)	3.60 (2.40)	3.61 (2.33)	3.55 (2.40)	3.61 (2.33)	3.64 (2.34)	3.67 (2.27)
Sleep duration, hours/day	7.20 (1.11)	7.17 (1.13)	7.18 (1.29)	7.22 (1.26)	7.15 (1.28)	7.17 (1.18)	7.17 (1.16)	7.15 (1.10)
Liver profile								
Alanine aminotransferase (ALT), U/L	24.03 (13.66)	24.75 (12.53)	26.60 (17.20)	25.78 (15.63)	36.28 (30.68)	23.99 (13.45)	25.59 (16.77)	23.60 (14.20)
Alkaline phosphatase (ALP), U/L	84.98 (27.46)	87.80 (45.72)	87.98 (28.85)	86.01 (24.23)	99.09 (54.37)	87.65 (26.65)	87.07 (30.59)	83.10 (25.40)
Aspartate aminotransferase (AST), U/L	26.63 (11.25)	27.36 (11.44)	29.16 (17.32)	27.74 (10.15)	39.27 (28.86)	26.77 (11.18)	28.23 (15.16)	26.20 (10.50)
Gamma glutamyltransferase (GGT), U/L	40.99 (47.19)	44.70 (53.28)	51.48 (67.23)	43.20 (52.30)	109.06 (156.97)	43.76 (60.60)	48.37 (69.40)	37.10 (40.80)
Total protein, g/L	72.18 (4.06)	72.27 (4.11)	72.16 (4.12)	72.26 (4.23)	73.08 (4.62)	72.41 (4.12)	72.27 (4.13)	72.60 (4.09)
Albumin, g/L	44.96 (2.59)	44.97 (2.64)	44.78 (2.64)	44.77 (2.63)	44.30 (2.88)	44.87 (2.61)	44.86 (2.65)	45.30 (2.62)
Total bilirubin, umol/L	9.33 (4.37)	9.05 (4.98)	9.13 (3.96)	9.06 (4.08)	10.35 (5.81)	8.74 (4.15)	9.29 (4.55)	9.16 (4.44)
Direct bilirubin, umol/L	1.88 (0.84)	1.86 (1.22)	1.90 (0.87)	1.84 (0.76)	2.34 (1.55)	1.78 (0.73)	1.91 (0.95)	1.84 (0.85)
Cardiovascular health								
Apolipoprotein A1, g/L	1.52 (0.27)	1.52 (0.28)	1.48 (0.28)	1.45 (0.26)	1.49 (0.29)	1.52 (0.28)	1.51 (0.28)	1.54 (0.27)
Apolipoprotein B, g/L	1.04 (0.24)	1.03 (0.25)	1.01 (0.25)	1.01 (0.24)	0.95 (0.26)	1.03 (0.24)	1.03 (0.25)	1.03 (0.24)
C-reactive protein, mg/L	2.85 (4.59)	2.94 (4.60)	3.33 (5.61)	3.14 (4.98)	3.45 (4.62)	3.04 (0.10)	2.99 (4.84)	2.53 (4.23)
Lipoprotein(A), nmol/L	44.86 (49.95)	45.67 (50.09)	44.91 (50.45)	45.42 (48.35)	40.34 (44.24)	44.70 (49.6)	44.57 (49.55)	44.6 (49.2)
Hdl cholesterol, mmol/L	1.42 (0.39)	1.40 (0.39)	1.34 (0.38)	1.31 (0.35)	1.34 (0.40)	1.41 (0.39)	1.40 (0.39)	1.45 (0.38)
Ldl cholesterol, mmol/L	3.54 (0.91)	3.51 (0.91)	3.41 (0.89)	3.43 (0.85)	3.24 (0.96)	3.53 (0.89)	3.50 (0.91)	3.56 (0.97)
Total cholesterol, mmol/L	5.67 (1.20)	5.63 (1.21)	5.46 (1.20)	5.45 (1.14)	5.26 (1.30)	5.65 (1.17)	5.61 (1.22)	5.69 (1.14)
Triglycerides, mmol/L	1.87 (1.09)	1.93 (1.11)	1.97 (1.07)	1.97 (1.06)	1.99 (1.26)	1.87 (1.02)	1.89 (1.10)	1.74 (1.03)
Renal profile								
Creatinine, umol/L	74.61 (24.64)	7.41 (16.74)	76.66 (15.44)	77.70 (16.76)	74.71 (16.82)	72.72 (16.97)	74.47 (21.10)	72.3 (17.9)
Cystatin C, mg/L	0.94 (0.22)	0.95 (0.18)	0.97 (0.17)	0.97 (0.18)	0.99 (0.19)	0.94 (0.16)	0.95 (0.20)	0.90 (0.17)
Urea, mmol/L	5.53 (1.55)	5.57 (1.47)	5.45 (1.44)	5.58 (1.36)	5.54 (1.57)	5.50 (1.34)	5.52 (1.51)	5.38 (1.38)
Urate, umol/L	324.71 (83.36)	328.50 (79.81)	337.40 (81.07)	339.03 (83.48)	333.31 (86.05)	319.00 (80.05)	327.07 (83.39)	309.00 (80.30)
Hormones								
Insulin-like growth factor 1 (IGF-1), nmol/L	20.89 (5.74)	20.43 (5.40)	20.25 (6.10)	20.63 (5.56)	17.13 (6.92)	20.66 (5.94)	20.42 (5.89)	21.5 (5.67)
Oestradiol, pmol/L	391.32 (412.80)	326.08 (279.44)	308.03 (370.01)	307.13 (250.03)	276.73 (165.39)	409. 10 (476.70)	362.69 (380.97)	463.00 (430.00)
Sex hormone binding globulin (SHBG), nmol/L	49.48 (26.15)	49.43 (25.45)	48.13 (24.92)	47.94 (25.27)	58.01 (31.01)	50.86 (28.51)	50.21 (26.76)	51.30 (27.60)
Testosterone, nmol/L	7.64 (5.91)	7.39 (5.88)	9.43 (5.59)	9.15 (5.64)	9.20 (6.66)	6.73 (6.13)	7.84 (6.01)	6.65 (6.07)
Bone-related markers								
Calcium, mmol/L	2.38 (0.10)	2.38 (0.11)	2.37 (0.09)	2.37 (0.09)	2.38 (0.11)	2.38 (0.10)	2.38 (0.10)	2.38 (0.09)
Phosphate, mmol/L	1.15 (0.16)	1.16 (0.16)	1.15 (0.16)	1.13 (0.17)	1.12 (0.17)	1.17 (0.16)	1.15 (0.16)	1.16 (0.16)
25-Hydroxyvitamin D (25(OH)D), nmol/L	48.50 (21.42)	48.39 (21.28)	47.37 (21.24)	47.54 (20.99)	46.01 (21.56)	48.80 (21.86)	48.20 (21.48)	48.40 (21.10)
Rheumatoid factor, IU/ml	25.50 (21.37)	22.71 (17.05)	24.52 (19.79)	26.67 (22.51)	24.80 (20.21)	20.68 (15.97)	24.84 (21.01)	24.50 (19.80)
Glucose control								
Glycated hemoglobin (Hba1C), %	5.53 (0.66)	5.63 (0.72)	5.64 (.076)	5.65 (0.79)	5.82 (1.01)	5.56 (0.68)	5.59 (0.74)	5.45 (0.62)
Hematology								
Basophil count, 10^9^ cells/L	0.035 (0.06)	0.035 (0.06)	0.037 (0.048)	0.035 (0.05)	0.035 (0.05)	0.034 (0.04)	0.035 (0.06)	0.034 (0.05)
Eosinophil count, 10^9^ cells/L	0.178 (0.132)	0.186 (0.146)	0.193 (0.171)	0.193 (0.159)	0.189 (0.12)	0.184 (0.14)	0.183 (0.14)	0.175 (0.14)
Erythrocyte count, 10^9^ cells/L	4.55 (0.41)	4.54 (0.42)	4.61 (0.42)	4.63 (0.41)	4.53 (0.42)	4.52 (0.39)	4.55 (0.41)	4.53 (0.42)
Lymphocyte count, 10^9^ cells/L	1.97 (0.85)	1.96 (0.65)	1.97 (0.77)	2.00 (0.72)	1.99 (0.81)	1.99 (0.65)	1.96 (0.63)	1.96 (0.78)
Monoctye count, 10^9^ cells/L	0.50 (0.20)	0.50 (0.23)	0.55 (0.32)	0.53 (0.30)	0.52 (0.24)	0.49 (0.19)	0.50 (0.22)	0.47 (0.20)
Neutrophil count, 10^9^ cells/L	4.35 (1.39)	4.43 (1.51)	4.62 (1.53)	4.47 (1.49)	4.29 (1.46)	4.32 (1.37)	4.38 (1.43)	4.22 (1.40)
Platelet count, 10^9^ cells/L	248.78 (58.64)	248.21 (57.40)	247.31 (62.35)	248.64 (60.40)	222.88 (68.79)	252.9 (62.28)	246.90 (60.77)	253.00 (59.40)
Hemoglobin, g/dL	14.37 (1.21)	14.34 (1.22)	14.63 (1.23)	14.56 (1.25)	14.46 (1.30)	14.22 (1.19)	14.39 (1.24)	14.20 (1.25)
White blood cell count, 10^9^ cells/L	7.04 (1.86)	7.11 (1.91)	7.38 (2.00)	7.24 (1.86)	7.03 (1.92)	7.02 (1.75)	7.06 (1.81)	6.87 (1.83)

Mean (standard deviation) is presented for continuous variables.

^a^Mean (SD) values and n (percentages) are reported for continuous and categorical variables, respectively.

^b^Other GI cancer includes small intestine cancer, cancer of anus and anal canal, gallbladder cancer, cancer of other and unspecified parts of biliary tract, and cancer of other digestive organs.

We employed univariable and multivariable logistic regression analyses of phenomic features against outcomes of interest (first/initial diagnoses of disease) in this study, similar to studies conducted by Gausman et al.^32^ and Kang et al.^33^. Initially, univariable analysis using logistic regression was applied to determine the significant risk factors for the outcomes of interest. The Benjamini–Hochberg correction was implemented to control the False Discovery Rate (FDR) and mitigate the risk of false positives. The Benjamini–Hochberg correction is a widely accepted method for controlling the FDR, offering a more balanced approach than the Bonferroni correction. Unlike the Bonferroni correction, which is known for its conservative nature and increased likelihood of false negatives, the Benjamini–Hochberg procedure allows for a more nuanced control of the error rate. By controlling the FDR instead of the Family-Wise Error Rate (FWER), the BH procedure provides a good balance between identifying true positives and limiting false positives.

Since the cohort data analysed was large, besides relying on the p-value, odds ratios (OR) of more than 2.0 and 1.5 for categorical and numerical variables respectively was fixed to screen the significant and important risk factors^34^. Univariable logistic regression results for all variables can be found in Supplementary Table 1. All the significant variables in the univariable logistic regression for each GI cancers and total GI cancers were listed in Table 2. Next, multivariable logistic regression based on the variables identified in Table 2 were conducted. One important criterion for model selection in logistic regression is the assumption that the independent variables should not correlate with each other. Therefore, variable selection in multivariable logistic regression was performed in a way that the variables of the same category will be chosen based on the variable with the highest odds ratio in univariable logistic regression. The odds ratio with respective 95% confidence interval and p-values for each variable were reported in Table 3. The analyses was conducted without prior considerations of potential causal pathway, stratification based on socio-demographic and lifestyle factors to avoid analysis bias.

**Table 2:** Factors associated with GI cancers in the UKB cohort based on univariable logistic regression.

Variables	Variables	Colorectal cancer	Pancreatic cancer	Oesophageal cancer	Gastric cancer	Liver cancer	All GI cancers
Sociodemographic	Race^a^	√	√	√		√	√
	Gender^a^			√	√	√	
	Alcohol drinking status^a^			√			
	Smoking status^a^			√		√	
Anthropometric measurement	Body mass index^a^			√	√	√	
Biochemical markers	Apolipoprotein A1			√	√	√	
	Apolipoprotein B			√		√	
	Calcium			√	√	√	
	Cystatin C	√	√	√	√	√	√
	Phosphate			√	√	√	
	HDL cholesterol			√	√	√	
	LDL cholesterol					√	
Hematological markers	Basophil count			√			
	Eosinophil count		√	√	√	√	
	Erythrocyte count			√	√		
	Monocyte count			√	√	√	

Based on p-value < 0.05 and Odds Ratio (OR) ≥ 1.5 for numerical variables and OR ≥ 2 for categorical variables.

^a^Analysed as categorical variables.

**Table 3:** Factors associated with total GI cancers and top five GI cancers in the UK Biobank cohort (using multiple logistic regression).

GI cancer	Variables	Adj. *OR*	(95% CI *OR*)	p-value
Total GI cancers	Cystatin C	2.43	(2.23, 2.64)	< 0.001
	Race			
	White	2.22	(1.69, 2.74)	< 0.001
	Asian (ref)	1.00	–	
Colorectal cancer	Cystatin C	2.11	(1.92, 2.32)	< 0.001
	Race			
	White	2.54	(1.93, 3.34)	< 0.001
	Asian (Ref)	1.00	–	–
Pancreatic cancer	Cystatin C	2.15	(1.85, 2.49)	< 0.001
	Eosinophil count	1.41	(1.02, 1.95)	0.035
	Race			
	White	2.51	(1.42, 4.43)	0.002
	Asian (ref)	1.00	–	–
Oesophageal cancer	Apolipoprotein A1	0.72	(0.56, 0.94)	0.017
	Calcium	0.37	(0.18, 0.76)	0.007
	Cystatin C	2.15	(1.81, 2.54)	< 0.001
	Monocyte count	1.58	(1.41, 1.79)	< 0.001
	Body mass index			
	Underweight	2.60	(1.27, 5.33)	0.009
	Normal weight (ref)	1.00	–	–
	Overweight	1.57	(1.32, 1.87)	< 0.001
	Obese	1.84	(1.52, 2.23)	< 0.001
	Race			
	White	3.92	(1.75, 8.78)	< 0.001
	Asians (ref)	1.00	–	–
	Smoking status			
	Never (ref)	1.00	–	–
	Previous	2.01	(1.73, 2.33)	< 0.001
	Current	3.01	(2.52, 3.61)	< 0.001
Gastric cancer	Cystatin C	1.97	(1.64, 2.38)	< 0.001
	HDL cholesterol	0.71	(0.55, 0.91)	0.007
	Monocyte count	1.41	(1.20, 1.67)	< 0.001
	Body mass index			
	Underweight	2.35	(0.95, 5.82)	0.064
	Normal weight (ref)	1.00	–	–
	Overweight	1.23	(1.01, 1.50)	0.039
	Obese	1.56	(1.26, 1.93)	< 0.001
	Gender			
	Male	2.35	(1.97, 2.80)	< 0.001
	Female (ref)	1.00	–	–
Liver cancer	Apolipoprotein B	0.27	(0.19, 0.39)	< 0.001
	Cystatin C	2.30	(1.95, 2.70)	< 0.001
	Monocyte count	1.36	(1.11, 1.67)	0.003
	Phosphate	0.36	(0.22, 0.58)	< 0.001
	Gender			
	Female (ref)	1.00	–	–
	Male	1.66	(1.40, 1.97)	< 0.001
	Smoking status			
	Never (ref)	1.00	–	–
	Previous	1.71	(1.44, 2.02)	< 0.001
	Current	1.88	(1.48, 2.38)	< 0.001

*Adj. OR* adjusted odds ratio.

Data extraction and processing was conducted on the UK Biobank Research Analysis Platform (RAP) through Jupyterlab and Jamovi^35^. All analyses were carried out using Jamovi^35^.

### Ethics approval

The UK Biobank received ethical approval from the National Information Governance Board for Health and Social Care and the National Health Service North West Centre for Research Ethics Committee. The study was conducted in accordance with the Declaration of Helsinki. This study was also approved by the Malaysia Medical Research and Ethics Committee (NMRR ID-23-00931-SPO).

### Consent to participate

All participants provided written informed consent prior to recruitment.

## Results

The analysis included a total of 441,141 participants, of which 7952 (1.8%) were incident GI cancer cases and 433,189 were healthy controls. Among the 7952 participants with GI cancers, there were 11,563 total GI cancers recorded. A subset of participants with GI cancers and cancer(s) other than GI cancers (1468 participants) were not included in the subsequent logistic regression analysis.

Table 1 shows the characteristics of healthy controls, participants who developed GI cancers by total GI cancers and top 5 GI cancers, in the order of colorectal cancer (n = 5436), pancreatic cancer (n = 1286), oesophageal cancer (n = 1146), gastric cancer (n = 878) and liver cancer (n = 755). Comparing with the control group, the GI cancer group was older when they were recruited, consisted of more males, had higher BMI, and a higher proportion of participants being current or previous smokers. Participants with GI cancer were followed up for an average of 7.5 years before they were diagnosed with GI cancer, while healthy controls were followed up for a mean duration of 8.5 years.

Table 1 illustrates the significant variables that are associated with each GI cancer and total GI cancers, based on univariable logistic regression. Race and cystatin C were significantly associated with total GI cancers. Cystatin C was also significantly associated across each type of GI cancer whereas race is associated with all GI cancers except gastric cancer. There seemed to be gender differences in oesophageal, gastric and liver cancers, with men having higher risk in developing these cancers. Lifestyle factors (alcohol drinking and smoking status) were also associated with some GI cancers (oesophageal cancer for both factors and liver cancer for smoking status). Anthropometric measurement (body mass index classification) was also associated with oesophageal, gastric and liver cancers.

In terms of biochemical markers, several cardiovascular health markers (apolipoproteins A and B, HDL cholesterol, LDL cholesterol) on top of ionized calcium and phosphate were associated with some GI cancers. There were also hematological markers, including basophil, eosinophil, erythrocyte and monocyte that are found to be associated with some GI cancers.

These variables that were significantly correlated to GI cancers based on univariable logistic regression were further analysed in multivariable logistic regression (Table 3). Against total GI cancers, an increase of 1 mg/L cystatin C multiplied the odds of getting total GI cancers by 2.43 times. Analysis also found that compared to Asians, participants of White race had a 2.22 times higher risk of getting GI cancers.

When we looked at each individual GI cancer, cystatin C remained significantly associated with the cancers with an adjusted odds ratio of at least 1.97. Cystatin C and White participants had a higher risk of getting colorectal cancers (adjusted OR of 2.11 and 2.54 respectively), which was the top one GI cancer in the UK Biobank cohort. Participants with a diagnosis of pancreatic cancer are associated with higher cystatin C (adjusted OR 2.15), eosinophil count (adjusted OR 1.41) and White ancestry (adjusted OR 2.51 compared to Asians). For oesophageal cancer, besides cystatin C and of White race, lower Apolipoprotein A1, higher monocyte count and lower ionized serum calcium were associated with higher risk of getting oesophageal cancer. Compared to normal weight participants, those who were underweight (adjusted OR 2.75), overweight (adjusted OR 1.44) and obese (adjusted OR 1.84) were associated with oesophageal cancer. Previous and current smokers were also found have higher risk (adjusted OR 1.82 and 2.77 respectively) of getting oesophageal cancer. On the other hand, gastric cancer was associated with male subjects, those with BMI other than normal weight, lower HDL cholesterol and higher monocyte count. Lastly, in addition to cystatin C, apolipoprotein B, phosphate, monocyte count, males and those of smoking history were associated with liver cancer.

## Discussion

In this large UK Biobank cohort study of a total of 7952 incident GI cancer cases, we aimed to investigate the associations between the phenomic features and GI cancers to better understand the molecular pathogenesis of GI tract cancers. The analysis included a total of 441,141 participants in the study, of whom 7952 (1.8%) were incident cases of GI cancer and 433,189 were healthy controls. The results demonstrated significant associations between certain variables and different types of GI cancers, providing valuable insights into the risk factors and potential biomarkers associated with these cancers. The characteristics of the GI cancer group were substantially different from those of the control group, with the GI cancer group being older, predominantly male, having a higher BMI, and containing a greater proportion of current or former smokers.

The distribution of the top five GI cancers observed in this UK Biobank cohort was found to be consistent with global trends^3,36^. Colorectal cancer (47.01% of the total GI cancer cases) emerged as the most common GI cancer, followed by pancreatic cancer (11.12%), oesophageal cancer (9.91%), gastric cancer (7.59%), and liver cancer (6.50%). This pattern aligns with previous studies and reflects the epidemiology of GI cancers on a global scale, indicating the generalizability of the findings from this cohort to broader populations. As the top five GI cancers in the UK Biobank cohort represented 82% of the entire GI cases, subset analysis focuses on the five out of nine major GI cancer categories.

One of the notable findings in this study was the consistent association of cystatin C and race with each type of GI cancer. Cystatin C, a biomarker related to kidney function^37,38^, was found to be consistently raised and associated with all GI cancers in this cohort. Participants with higher cystatin C levels exhibited an increased risk of developing GI cancers, suggesting its potential as a prognostic biomarker. This finding is corroborated in previous literature^39–42^. Cystatin C exerts a series of complex effects that may result in either an inhibition or a promotion of tumour cell growth and dissemination, as demonstrated by previous research^39,40^. A recent study discovered a novel mechanism of mast cells inducing endoplasmic reticulum stress in which Cystatin C mediates tumor inhibition during colorectal cancer development^41^ This function of Cystatin C in cancer cells has never been reported and may lead researchers one step closer to understanding the molecular pathogenesis of GI cancers in relation to cystatin C.

Additionally, race was found to be significantly associated with total GI cancers, with Whites having a higher risk compared to Asians. The influence of race is also evident in subsets colorectal, pancreatic and oesophageal cancers in this study. Epidemiological studies have examined the association between race, specifically White and Asian populations, and gastrointestinal malignancies, including colorectal, pancreatic, esophageal, gastric, and liver cancer^3,43–46^. Similarly, results showed that gender played a role in the difference in GI cancer incidence, particularly gastric and liver cancers, males having 2.8, 2.4 and 1.7 times more likely to get the cancers respectively. This finding is in line with current literature^45,47,48^. While acknowledging the limited representation of Asians in the UK Biobank cohort, the study emphasizes that phenotypic feature identification is its main goal in relation to GI malignancies. Importantly, the study emphasized that the relatively small number of Asians in the cohort should not undermine the robustness of the scientific inferences drawn regarding associations between exposures and health conditions.

In addition to sociodemographic characteristics, lifestyle factor particularly smoking status was proven to be associated with certain GI cancers, including liver and oesophageal cancers. Smoking status still remained a significant factor in the multivariable logistic regression analysis for liver cancer. Interestingly, exposure to smoking (including those who had stopped smoking) consistently increased the risk of developing GI cancers. This is supported and demonstrated in other studies as well^45,47,49^. Cancer incidence and mortality rate variations are influenced by several factors, including genetic, environmental, lifestyle, and socioeconomic variables^43–45,50^.

Anthropometric measurement (body mass index classification) showed associations with oesophageal and gastric cancers. In line with the work of other researchers, we demonstrated *U*-shape relationship between BMI and the three cancers^51–53^. This abundant evidence of excess body weight over the past few decades indicates an emphasis on lipid metabolism and mechanisms involved in malignancies^26,51–54^. As demonstrated in this study, apolipoprotein A1, apolipoprotein B and HDL cholesterol were associated with oesophageal, gastric and liver cancers. Studies have suggested that apolipoproteins play critical roles in malignancies including GI cancers. Low apolipoprotein A1 level is linked to a high cancer risk, systemic inflammatory response and poorer survival in some cancers, including oesophageal squamous cell carcinoma^55–58^. This is in accordance with our study findings. Apolipoprotein A1 is a protein component of HDL cholesterol. Similar to apolipoprotein A1, HDL cholesterol is inversely associated with cancers, as demonstrated in the subset gastric cancer in this study. One of the proposed mechanisms of the opposing role in tumorigenesis of HDL cholesterol is its modulation of cell cycle entry and apoptosis through the mitogen-activated protein kinase-dependent (MAPK) pathway^59^. A Korean cross-sectional study also reported the association between reduced HDL/apolipoprotein A1 levels and an increased risk of colorectal cancer^60^. Emerging evidence suggests that the apolipoprotein A1/HDL axis, involved in lipid metabolism, is dysregulated in cancer. mRNA levels of apolipoprotein A1 were lower in hepatocellular carcinoma compared to normal liver tissue, the primary source of apolipoprotein A1, as determined by Oncomine database microarray data^61^. In hepatocellular carcinoma, the mechanisms underlying the transcriptional repression of apolipoprotein A1 remain obscure.

However, this result is consistent with previous reports of decreased apolipoprotein A1 protein levels in malignant liver tissue and hepatocellular carcinoma patient serum^62,63^. The decrease in apolipoprotein A1 transcription, intracellular and secreted apolipoprotein A1, and circulating HDL levels in hepatocellular carcinoma suggests that this pathway may have a tumor-suppressing function^61^. Several studies have discovered associations between serum apolipoprotein A1/HDL levels and various aspects of the natural progression of various cancer types^56,59,64,65^. Consistent with the study findings, high apolipoprotein B level was suggested as a risk factor for liver cancer; it is associated with poorer survival post surgery and a larger tumour size^66^. More in-depth exploration of the genetic information of apolipoproteins may indicate liver malignancy and thus should be further researched on. Mutations of apolipoprotein B is reported to account for almost 10% of all genetic mutations^66^. Specifically, a non-oncogenetic mutation of apolipoprotein B is observed, which can result in apolipoprotein B inactivation and is associated with the overexpression of oncogenic regulators and the downregulation of tumour suppressors, resulting in poorer survival outcomes. It is hypothesised that mutations that render apolipoprotein B inactive are preferred in tumorigenesis in order to provide more energy for cancer metabolism^55,65^.

Multivariable logistic regression demonstrated that ionized serum calcium level was inversely associated with the risk of oesophageal cancer (adjusted OR = 0.37, 95% CI 0.18–0.74; p-value = 0.005). This is in line with studies that established the significance of calcium intake, in particular, as a potential effect modifier of the association between calcium and diseases including GI tract neoplasia^67–69^. Increasing dietary calcium intake was associated with lower risk of oesophageal cancer^67–69^. There seems to be inconsistent findings on the relationship between serum calcium and risk of cancer in current literature. The Swedish AMORIS study exploring GI cancers specifically oesophageal, stomach and CRC cancers, showed positive association between albumin-adjusted serum calcium and risk of these GI cancers^70^. Nevertheless, a study exploring two large European prospective cohorts (including the UK Biobank) corroborated our study findings on ionized serum calcium level and risk of liver and colorectal cancer^71^.

The different direction of the association between the UK Biobank and EPIC cohorts, and the AMORIS study was attributed to differences in study design and the degree of adjustment for confounding variables^71^. It is worthwhile to discuss on this study’s focus on serum calcium measurement rather than dietary calcium intake. Serum calcium indicates extracellular calcium homeostasis and is mainly regulated by vitamin D and parathyroid hormone. Consequently, abnormalities in serum calcium level may reflect an error in its regulation pathways instead of dietary calcium deficiency. This may result in distinct associations between calcium in the diet and serum and carcinogenesis^70–72^. Besides calcium, phosphate is also found to be inversely associated with liver cancer (adjusted OR = 0.36; 95% CI 0.22–0.58; p-value = 0.001). There is little research on phosphate and cancers, with inconsistent trends among the studies and/or cancers^54,73,74^. It is accepted that altered levels of phosphate have been linked to the onset of cancer, but with uncertainties on the pathophysiology behind it. More in-depth studies are warranted to better understand the positive and inverse correlation observed between calcium and phosphate levels, and the risk of cancers. This will shed light on the involvement of calcium and phosphate metabolism, and potentially related important hormonal factors and cancer.

Additionally, hematological markers including monocyte and eosinophils were related to some GI cancers. Monocytes and eosinophils are a type of white blood cell. Interestingly, there are scarce research on the association of eosinophils and monocytes in GI cancer. Despite that, the value of immune-related markers in cancers are acknowledged. Previous studies focused mainly on pre-operative values of these circulating cells, however, changes in the immune profile may occur months or years prior to cancer diagnosis due to its role in the etiopathegenesis of tumours^75^. White blood cells were previously found to be associated with increased risk of colorectal, lung and breast cancer^76^. Preclinical data showed that eosinophils have both pro-tumorigenic and anti-tumorigenic properties, via direct and indirect mechanisms. This varying outcomes in different studies imply that the role of eosinophils and their mediators may differ depending on the cancer type^77–79^.

These findings provide valuable insights into the associations between various factors and GI cancers within the UK Biobank cohort. The identification of significant associations contribute to our understanding of the underlying mechanisms and risk factors involved in the development of GI cancers. The consistent association of cystatin C with different types of GI cancers suggest its potential as a promising biomarker for early detection and risk stratification. The findings from this study will guide our subsequent way forward to explore the whole exome sequencing data in GI cancers within the UK Biobank. This will promote a multi-omic methodology to help characterize GI cancers and associated phenomic features. Specifically, variants within the exome region of the genome, which is responsible for encoding proteins, can serve as valuable indicators for the identification of genetic variants that are highly relevant to drug discovery^80^.

Notable strengths of this study include its prospective study design involving a large sample size, a lengthy follow-up period and evaluation of a comprehensive list of covariates. In addition, all biochemistry markers were measured using well-established and validated methods, ensuring accuracy and reliability throughout the study. This study, is however, not without its limitations. Despite UK Biobank not being suitable for determining universally applicable rates of disease prevalence and incidence, its substantial size and diverse exposure measures allow for valid scientific inferences on associations between exposures and health conditions. Such assessments can be widely generalizable and do not necessitate participants to be representative of the population at large^19,81,82^. In addition, this study focusses on the phenomic data involved on the pathogenesis of GI cancers, with aim to identify the potential phenomic feature(s) associated with the pathogenesis of GI cancers, and not to associate with incidence rate. Although the number is small, this is a cross sectional analyses of UK Biobank data, which still represent the largest database at present, and present findings are in accordance with previous studies looking into different health outcomes and their associations with race. In addition, the study relied on self-reported lifestyle data, which introduces the possibility of recall bias. To validate and expand upon these findings, additional research with diverse populations and rigorous data acquisition techniques is required. Besides, in terms of study data, no information was available regarding potential confounding variables such as vitamin D and/or calcium supplementation. Furthermore, we were unable to explain the effect of dietary calcium on gastrointestinal carcinogenesis, as suggested by biological studies (Supplementary Table 1).

In conclusion, this study identified several significant associations between various factors and GI cancers using the UK Biobank cohort. A marker Cystatin C emerged as a consistent biomarker associated with different types of GI cancers. Given the small proportion of Asians within the UK Biobank, the association between race and GI cancers requires further confirmation. The findings provide valuable insights into the potential diagnostic and therapeutic targets for GI cancers, emphasizing the importance of personalized approaches in cancer prevention, early detection, and treatment strategies. In order to provide more in-depth understanding of how these factors were associated with GI cancers and shed light on the molecular pathogenesis of GI cancers, future research should employ a multi-modal approach exploring the genomics and proteomics of the UK Biobank cohort. This will allow validation of the study findings and enhance understanding on the underlying mechanisms linking these factors to GI cancer development.

### Supplementary Information

Supplementary Table 1.

## Data Availability

The datasets generated during and/or analysed during the current study are available to bona fide researchers and can apply for access to the UK Biobank data at https://www.ukbiobank.ac.uk/enable-your-research/apply-for-access.
