# Supplementary material for: Identification of phenomic data in the pathogenesis of cancers of the gastrointestinal (GI) tract in the UK biobank
Source: Sci Rep. 2024 Jan 23;14:1997. doi: 10.1038/s41598-024-52421-9 (PMC10805853; doi:10.1038/s41598-024-52421-9)
Supplement: Supplementary file 1 — Supplementary Table 1. [file 41598_2024_52421_MOESM1_ESM.docx]

**Identification of phenomic data in the pathogenesis of cancers of the gastrointestinal (GI) tract: A UK biobank data analysis**

Shirin Hui Tan^1,2^, Catherina Anak Guan^1^, Mohamad Adam Bujang^1^, Wei Hong Lai^1^, Pei Jye Voon^3^, Edmund Ui Hang Sim^2^

^1^ Clinical Research Centre, Sarawak General Hospital, Ministry of Health Malaysia, Jalan Hospital, 93586, Kuching, Sarawak, Malaysia

^2^ Faculty of Resource Science and Technology, Universiti Malaysia Sarawak, 94300, Kota Samarahan, Malaysia

^3^ Department of Radiotherapy, Oncology and Palliative Care, Sarawak General Hospital, Ministry of Health Malaysia, Jalan Hospital, 93586, Kuching, Sarawak, Malaysia, Sarawak, Malaysia

**Supplementary Table 1** Univariable logistic regression results for each GI cancer and total GI cancer

| **GI Cancer** | **Variables** | **Adj. *OR*** | **(95% CI *OR*)** | **p-value** | **Adjusted p-value** |
| --- | --- | --- | --- | --- | --- |
| **Total GI Cancers** | Cystatin C | 2.29 | (2.12, 2.48) | <0.001 | <0.001 |
|  | Race |  |  |  |  |
|  | White | 2.17 | (1.77, 2.66) | <0.001 | <0.001 |
|  | Asian (Ref) | 1 | - |  |  |
| **Colorectal Cancer** | Cystatin C | 2.01 | (1.84, 2.19) | <0.001 | <0.001 |
|  | Race |  |  |  |  |
|  | White | 2.42 | (1.86, 3.15) | <0.001 | <0.001 |
|  | Asian (Ref) | 1 | - | - | - |
| **Pancreatic Cancer** | Cystatin C | 2.10 | (1.83, 2.40) | <0.001 | <0.001 |
|  | Eosinophil count | 1.51 | (1.13, 2.02) | 0.005 | 0.012 |
|  | Race |  |  |  |  |
|  | White | 2.47 | (1.43, 4.27) | 0.001 | 0.003 |
|  | Asian (Ref) | 1 | - | - | - |
| **Oesophageal Cancer** | Apolipoprotein A1 | 2.21 | (1.72, 2.82) | 0.017 | 0.017 |
|  | Apolipoprotein B | 1.54 | (1.19, 1.99) | 0.001 | 0.001 |
|  | Basophil count | 2.59 | (1.15, 5.83) | 0.021 | 0.029 |
|  | Eosinophil count | 1.76 | (1.38, 2.25) | <0.001 | <0.001 |
|  | Erythrocyte count | 1.65 | (1.43, 1.90) | <0.001 | <0.001 |
|  | Calcium | 2.8 | (1.42, 5.52) | 0.003 | 0.003 |
|  | Cystatin C | 2.29 | (2.02, 2.60) | <0.001 | <0.001 |
|  | HDL cholesterol | 2.19 | (1.83, 2.63) | <0.001 | <0.001 |
|  | Monocyte count | 1.76 | (1.60, 1.95) | <0.001 | <0.001 |
|  | Phosphate | 1.67 | (1.13, 2.45) | 0.010 | 0.011 |
|  | Gender |  |  |  |  |
|  | Male | 3.35 | (2.94, 3.83) | <0.001 | <0.001 |
|  | Female (Ref) | 1 | - | - | - |
|  | Alcohol drinking status |  |  |  |  |
|  | Never (Ref) | 1 | - | - | - |
|  | Previous | 2.3 | (1.60, 3.36) | <0.001 | <0.001 |
|  | Current | 1.14 | (0.85,1.58) | 0.388 | 0.388 |
|  | Body mass index |  |  |  |  |
|  | Underweight | 2.71 | (1.34, 4.84) | 0.002 | 0.002 |
|  | Normal weight (Ref) | 1 | - | - | - |
|  | Overweight | 1.72 | (1.47, 2.01) | <0.001 | <0.001 |
|  | Obese | 2.06 | (1.75, 2.43) | <0.001 | <0.001 |
|  | Race |  |  |  |  |
|  | White | 3.67 | (1.83, 7.35) | <0.001 | <0.001 |
|  | Asians (Ref) | 1 | - | - | - |
|  | Smoking status |  |  |  |  |
|  | Never (Ref) | 1 | - | - | - |
|  | Previous | 2.23 | (1.95, 2.55) | <0.001 | <0.001 |
|  | Current | 3.21 | (2.73, 3.78) | <0.001 | <0.001 |
| **Gastric Cancer** | Apolipoprotein A1 | 3.65 | (2.73, 4.88) | <0.001 | <0.001 |
|  | Calcium | 3.56 | (1.64, 7.71) | 0.001 | 0.002 |
|  | Cystatin C | 2.26 | (1.97, 2.60) | <0.001 | <0.001 |
|  | Eosinophil count | 1.73 | (1.32, 2.29) | <0.001 | <0.001 |
|  | Erythrocyte count | 1.81 | (1.54, 2.12) | <0.001 | <0.001 |
|  | HDL cholesterol | 3.02 | (2.43, 3.76) | <0.001 | <0.001 |
|  | Monocyte count | 1.65 | (1.46, 1.86) | <0.001 | <0.001 |
|  | Phosphate | 2.76 | (1.78, 4.29) | <0.001 | <0.001 |
|  | Body mass index |  |  |  |  |
|  | Underweight | 2.00 | (0.79, 4.12) | 0.095 | 0.095 |
|  | Normal weight (Ref) | 1 | - | - | - |
|  | Overweight | 1.55 | (1.30, 1.85) | <0.001 | <0.001 |
|  | Obese | 2.02 | (1.68, 2.43) | <0.001 | <0.001 |
|  | Gender |  |  |  |  |
|  | Male | 2.92 | (2.52, 3.39) | <0.001 | <0.001 |
|  | Female (Ref) | 1 | - | - | - |
| **Liver Cancer** | Apolipoprotein A1 | 2.02 | (1.50, 2.73) | <0.001 | <0.001 |
|  | Apolipoprotein B | 4 | (2.86, 5.57) | <0.001 | <0.001 |
|  | Cystatin C | 2.43 | (2.13, 2.78) | <0.001 | <0.001 |
|  | Eosinophil count | 1.62 | (1.16, 2.25) | 0.005 | 0.008 |
|  | HDL cholesterol | 2.2 | (1.76, 2.76) | <0.001 | <0.001 |
|  | LDL cholesterol | 1.57 | (1.43, 1.72) | <0.001 | <0.001 |
|  | Monocyte count | 1.54 | (1.33, 1.80) | <0.001 | <0.001 |
|  | Calcium | 2.47 | (1.07, 5.66) | 0.033 | 0.038 |
|  | Phosphate | 3.84 | (2.39, 6.16) | <0.001 | <0.001 |
|  | Gender |  |  |  |  |
|  | Female (Ref) | 1 | - | - | - |
|  | Male | 2.05 | (1.76, 2.37) | <0.001 | <0.001 |
|  | Smoking status |  |  |  |  |
|  | Never (Ref) | 1 | - | - | - |
|  | Previous | 1.84 | (1.57, 2.15) | <0.001 | <0.001 |
|  | Current | 2.05 | (1.65,2.53) | <0.001 | <0.001 |
|  | Body mass index |  |  |  |  |
|  | Underweight | 1.46 | (0.45, 3.44) | 0.458 | 0.458 |
|  | Normal weight (Ref) | 1 | - | - | - |
|  | Overweight | 1.28 | (1.06, 1.55) | 0.01 | 0.012 |
|  | Obese | 2.13 | (1.76, 2.56) | <0.001 | <0.001 |
